# Marine Natural Products Against Tuberculosis

**DOI:** 10.1100/tsw.2006.174

**Published:** 2006-07-21

**Authors:** Marcus Vinícius Nora De Souza

**Affiliations:** FioCruz-Fundação Oswaldo Cruz , Instituto de Tecnologia em Fármacos-Far Manguinhos, 21041-25650 - Rio de Janeiro-RJ, Brazil

**Keywords:** tuberculosis, marine natural products, drugs

## Abstract

Natural products represent an outstanding source of compounds that play an important role in the treatment of human diseases. Due to the importance of nature as a source of new drug candidates, the aim of this review is to highlight the marine natural products, which exhibit antituberculosis activity, discovered between 2000 and 2005.

## INTRODUCTION

Tuberculosis (TB) is an infection transmitted through the air caused by the bacterium *Mycobacterium tuberculosis* (identified in 1882 by R. Koch) that mainly affects and damages the lungs, but it may spread across almost any tissue or organ of the body[1]. TB became a worldwide problem between 1985 and 1992 when the number of TB cases increased, particularly in people infected with the HIV virus. Another problem that increased TB cases is multidrug-resistant tuberculosis (MDR TB) due to inconsistent or partial treatment. Because of these problems, the World Health Organization (WHO) declared TB a global health emergency in 1993. Statistics inform us that 8 million people became sick with TB last year with almost 3 million deaths that represent, in general, four deaths each minute. It is estimated that 30 million people will die in the next 10 years[2]. At present, the accepted treatment of TB involves a combination of the first-line drugs, isoniazid **1**, pyrazinamide **2**, ethambutol **3**, and rifampicin **4** (Fig. 1) given in combination over 6–9 months. The combinations are very important to prevent the emergence of multiple drug-resistant organisms, which would lead to an ineffective treatment[2].

In spite of the worldwide problem caused by TB, it has been nearly more than 30 years since a novel drug has been introduced to treat TB. In this context, marine natural products became a powerful source of new anti-TB compounds that will be covered in this review.

## MARINE NATURAL PRODUCTS

The interest and study of marine natural products against *M. tuberculosis* started with Andersen et al.[3] and Hamman et al.[4]. Andersen and coworkers reported the *in vitro* anti-TB activity of massetolide A **5** (MIC = 5–10 μg/ml) and viscosin **6** (MIC = 10–20 μg/ml)[3], cyclic depsipeptides isolated from a marine alga and tube worm, respectively (Fig. 2).

**Isoniazid (1)**
MIC = 0.1 mg/L

**Pyrazinamide (2)**
MIC = 100 mg/L

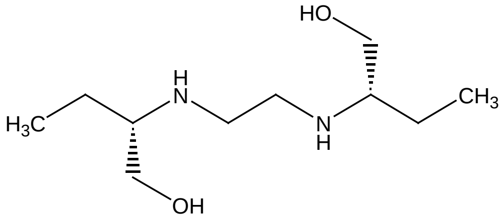

**Ethambutol (3)**
MIC = 2.5 mg/L

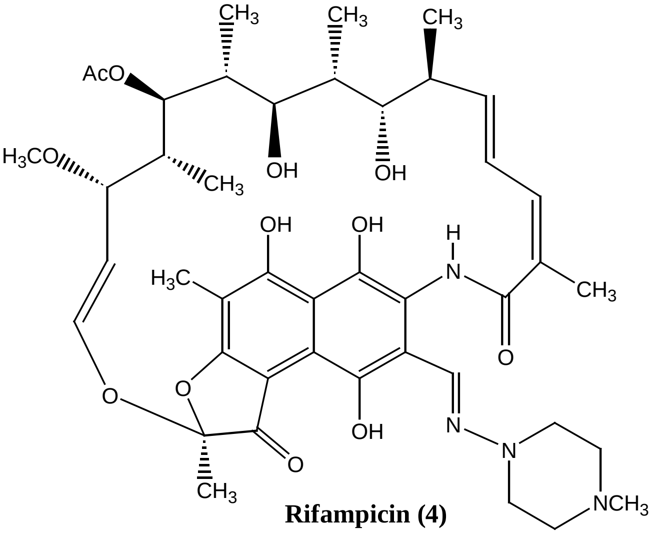

**Rifampicin (4)**
MIC = 2.0 mg/L

MIC - It is defined as the minimal inhibitory concentration of an antibiotic that inhibits a bacterium.

**FIGURE 1**

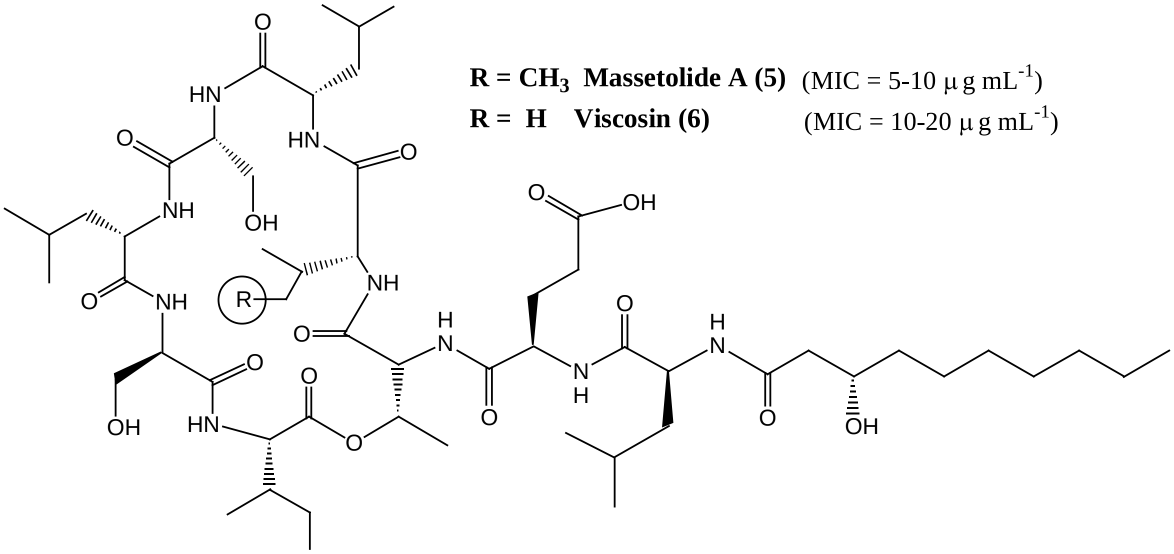

**R = $CH_3$ Massetolide A (5)** (MIC = 5-10 μg mL$^{-1}$)
**R = H Viscosin (6)** (MIC = 10-20 μg mL$^{-1}$)

**FIGURE 2**

Hamman and his group identified anti-TB activity in two known polypeptides isolated from the sacoglossan mollusk *Elysia rufescens*, Kahalalide A **7** and F **8** (Fig. 3)[4]. The first one has demonstrated important *in vitro* activity, inhibiting 83% the *M. tuberculosis* growth (H37Rv), at 12 μg/ml, and the second one, 67%, at the same concentration.

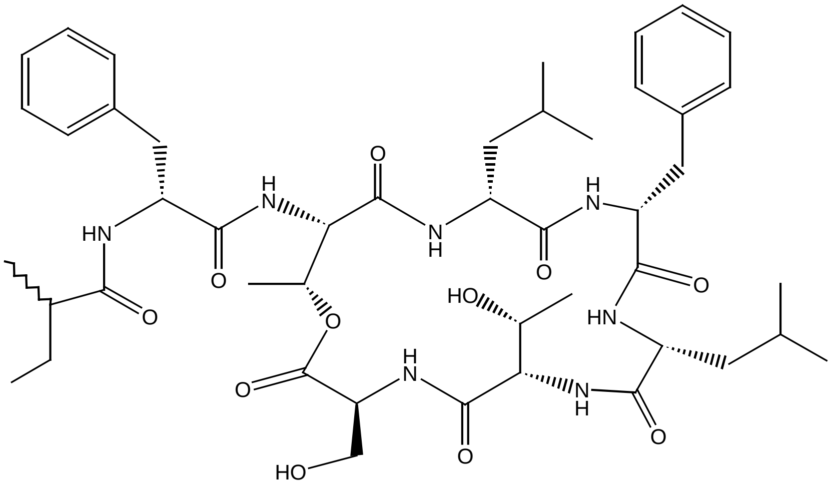

**Kahalalide A (7)** Inhibition activity = 83%
(MIC = 12.0 μg $mL^{-1}$)

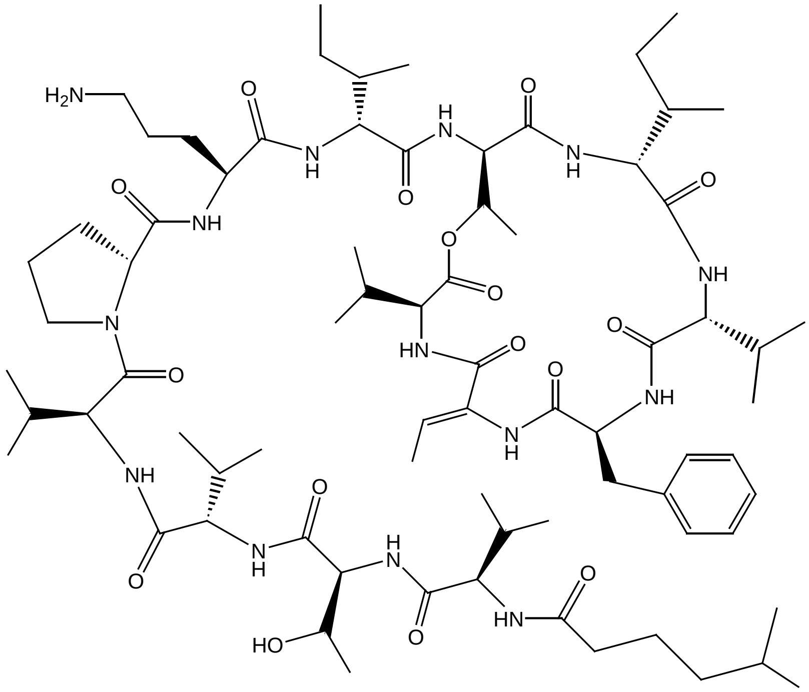

**Kahalalide F (8)** Inhibition activity = 67%
(MIC = 12.0 μg $mL^{-1}$)

**FIGURE 3**

Hamann and coworkers also made an important contribution by testing *in vitro* activities against *M. tuberculosis*, marine natural and semisynthetic molecules from different classes, in the total of 48 compounds[5]. The above work has shown potential candidates for new anti-TB agents and has indicated the importance of investigating marine natural products as a new source of antimycobacterial agents. As examples of the compounds tested by Hamann and his group, the C-19 hydroxysteroids linosterol **9**, nephalsterol B **10,** and C **11** that were isolated from red sea *Nepthea* sp. (Fig. 4) can be mentioned[6]. The natural product litosterol **9** and nephalsterol C **11** had MICs of 3.13 and 12.5 µg/ml inhibiting 90 and 96% of the growth of *M. tuberculosis* (H37Rv). However, nephalsterol B inhibited only 69% at the same concentration, which indicated that C-7 hydroxylation could reduce the activity. When C-7 hydroxyl was blocked by an acetate group as in the case of nephalsterol C **11** or when it was absent as in litosterol **9**, an improvement of the biological activity was observed. Hamann and his group were the first to identify antimycobacterial activity in this class of compounds.

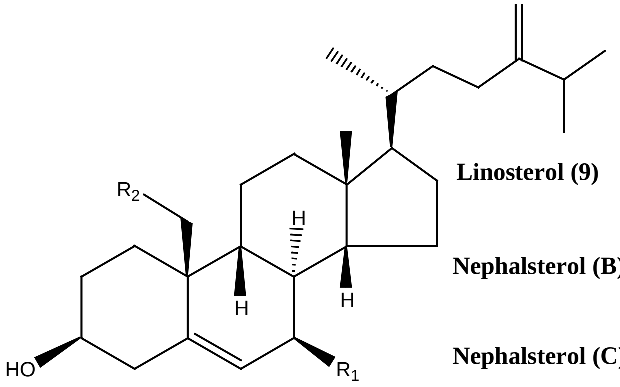

| | $R_1$ | $R_2$ | |
|---|---|---|---|
| **Linosterol (9)** | H | OH | Inhibition activity = 90% (MIC = 3.13 µ g $mL^{-1}$) |
| **Nephalsterol (B) (10)** | OH | OH | Inhibition activity = 69% (MIC = 12.5 µ g $mL^{-1}$) |
| **Nephalsterol (C) (11)** | OAc | OH | Inhibition activity = 96% (MIC = 12.5 µ g $mL^{-1}$) |

**FIGURE 4**

Hamman and coworkers have also isolated 27 diterpenes called cyanthiwigins from Jamaican sponge *Mymekioderma styx*[7], which have been tested against *M. tuberculosis.* However, the best result was the moderate activity of cyanthiwigin (C) **12**[8] with 50% of inhibition at 6.25 µg/ml (Fig. 5).

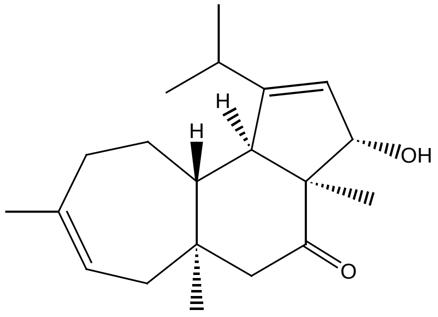

**Cyanthiwigins (C) (12)**
Inhibition activity = 50%
(MIC = 6.25 µ g $mL^{-1}$)

**FIGURE 5**

Another contribution was also done by Konig and coworkers who have tested 39 different marine natural products representative of different classes, such as terpenes, aliphatics, aromatics, alkaloids, and sterols[9]. In this context, 15 showed MICs of 32 μg/ml or less, and 11 had MICs of 16 μg/ml or less. The most promising compound reported in the Konig study was axisonitrile-3 **13** (MIC = 2.0 μg/ml) (Fig. 6) isolated from the sponge *Acanthella klethra* with no toxicity to Vero cells at concentrations < 200 μg/ml[9].

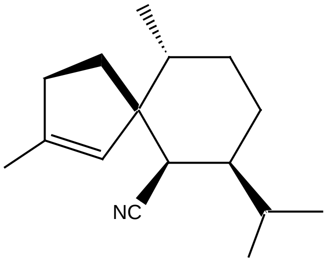

**Axisonitrile-3 (13)**
(MIC = 2.0 μ g $mL^{-1}$)

**FIGURE 6**

Manzamines are β-carbolines alkaloids from the Okinawan sponge genus *Haliclona* that were first reported in 1986 by Higa and coworkers[10]. This class of compounds possesses a diverse range of bioactivities, such as antibacterial[11], cytotoxic[12], and malaria activity[13]. In the search for new types of anti-TB agents, Hamman and coworkers have isolated and tested new manzanines from Indonesian sponges[14]. As examples, the two novel alkaloids called manadomanzamines A **14** and B **15** (Fig. 7) can be mentioned, isolated from Indonesian sponge *Acanthostrongylophora* sp. (Haploscilerida: Petrosiidae)[15] that exhibited strong activity against *M. tuberculosis* with MIC values of 1.9 and 1.5 μg/ml, respectively. These two alkaloids also exhibited significant activity against human immunodeficiency virus (HIV-1) with $EC_{50}$ values of 7.0 and 16.5 μg/ml, respectively, and moderate activity against several AIDS opportunist infections.

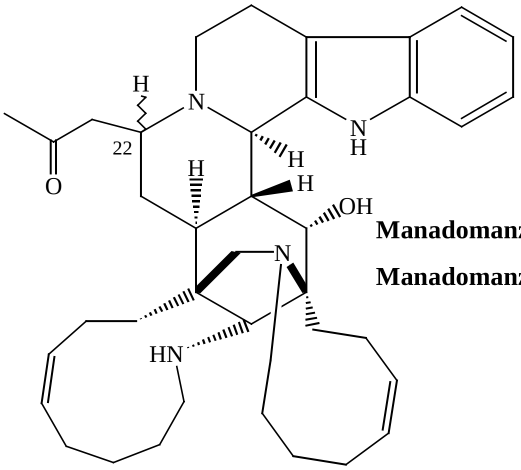

**Manadomanzamine A (14)**: 22 β – H (MIC = 1.9 μg $mL^{-1}$)
**Manadomanzamine B (15)**: 22 α– H (MIC = 1.5 μg $mL^{-1}$)

**FIGURE 7**

Rodrigues and coworkers have been an important contribution towards the search for new natural products against TB. They have purified and identified the natural products elisapterosin B **16**, elisabethin A **17**[16], cumbiasin A **18** and B **19**[17], and colombiasin A **20**[18] (Fig. 8). These compounds have similar carbocyclic skeletons and they were isolated from an extract of the West Indian corals gorgonian octocoral *Pseudopterogorgia elisabethae* Bayer (order Gorgonacea, family Gorgoniidae, phylum Cnideria) from the waters near San Andréas Islands, Colombia, showing potent activity against *M. tuberculosis*. The elisapterosin B **16** (Fig. 8) has been found to have potent inhibitory activity against *M. tuberculosis* $H_{37}Rv$ at a concentration of 12.3 μg/ml[16]. In the case of cumbiasin A **18** and B **19**, they display mild *in vitro* activity against $H_{37}Rv$, 6.25 and 12.5 μg/ml, respectively, and both compounds caused 17% growth inhibition of *M. tuberculosis*[18]. The pharmacological properties against *M. tuberculosis* of elisabethin A **17** and colombiasin A **20** (Fig. 8) have not yet been published.

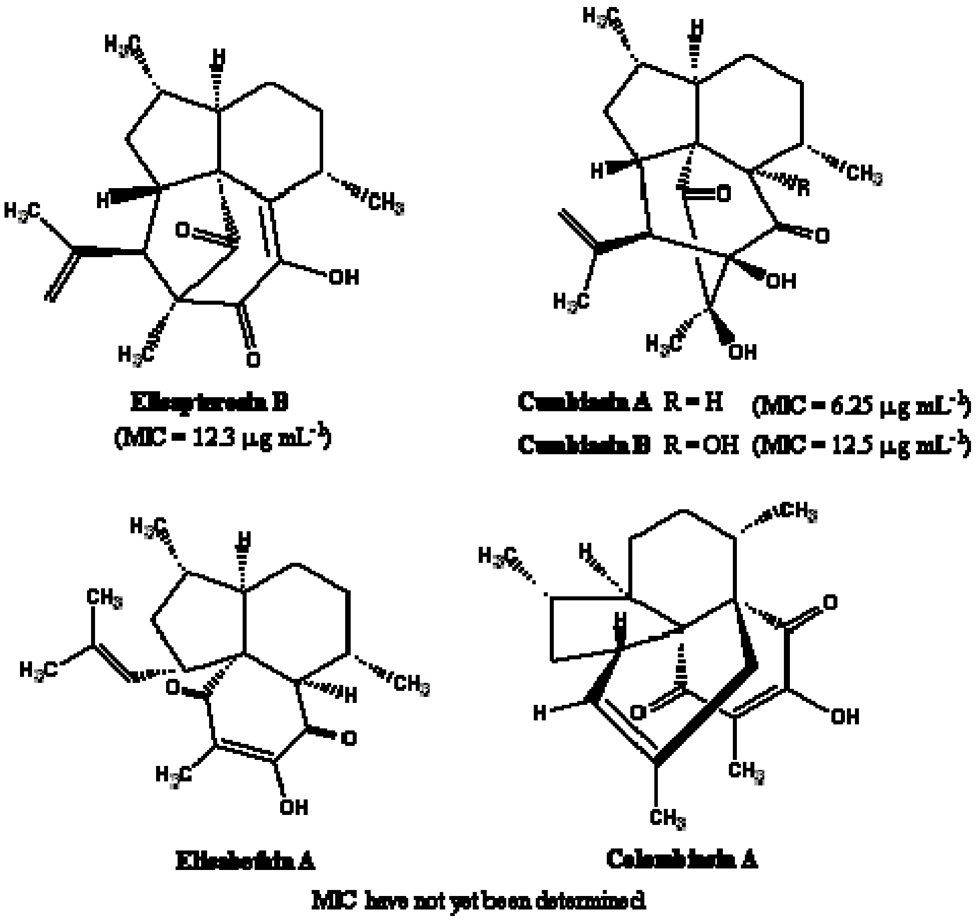

**FIGURE 8**

Other products that were identified by Rodrigues and coworkers from the same source of *P. elisabethae* also showed activity against *M. tuberculosis*. The three alkaloids, pseudopteroxazole **21**, homopseudopteroxazole **22**, and *seco*-pseudopteroxazole **23** (Fig. 9)[19] had potent to moderate inhibitory activity against *M. tuberculosis* $H_{37}Rv$ (97, 88, and 66%), respectively, at a concentration of 12.5 μg/ml. Another example is the two serrulatane-based diterpenes, erogorgiaene **24** and 7-hydroxyerogorgiaene **25**, which induced 96 and 77% inhibition of *M. tuberculosis* $H_{37}Rv$ at concentrations of 12.5 and 6.25 μg/ml, respectively. The biological results indicated that the oxazole moiety is not essential for activity[19].

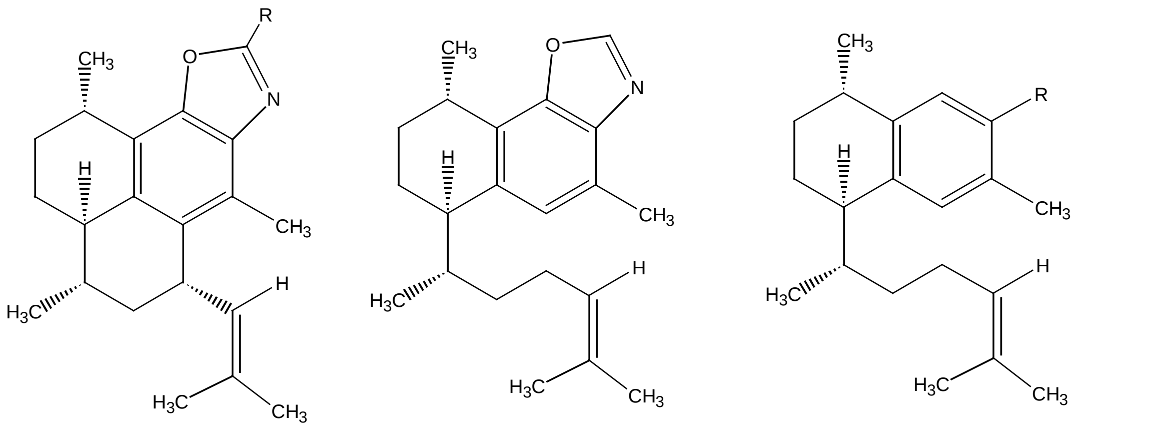

**Pseudopteroxazole (21)** R = H
Inhibition activity 97%
(MIC = 12.5 μ g $mL^{-1}$)

**Homopseudopteroxazole (22)**
R = $CH_2(CH_2)_3CH_3$
Inhibition activity 88%
(MIC = 12.5 μ g $mL^{-1}$)

***Seco*-pseudopteroxazole (23)**
Inhibition activity 66%
(MIC = 12.5 μ g $mL^{-1}$)

**Erogorgiaene (24)** R =H
Inhibition activity 97%
(MIC = 12.5 μ g $mL^{-1}$)

**7-Hydroxyerogorgiaene (25)** R = OH
Inhibition activity 77%
(MIC = 6.25 μ g $mL^{-1}$)

**FIGURE 9**

Due to their biological activity, complex molecular structure, and functionalization of the compounds present in *P. elisabethae*, many organic synthetic groups have been working in various synthetic strategies to prepare compounds of this class[20]. A good example is the elegant enantiospecific total synthesis of the pseudopteroxazole **21** (Scheme 1) made by Corey and Davidson[21]. They have used (S)-(-)-limonene **26** as starting material and after cyclic hydroboration and alkaline peroxide oxidation, the diol **27** was obtained. Under selective oxidation with sodium hypochlorite in aqueous acetic acid, this intermediate furnished the diastereoisomeric mixtures of hydroxy ketones **28**, which were enzymatically resolved, and after protection of the hydroxyl as the *tert*-butyl-diphenylsilyl ether (TBDPS), afforded the synthon **29** in 96% yield. The selective kinetic deprotonation of the intermediate using lithium diisopropylamide (LDA) and trapping chlorotrimethylsilane (TMSCl) furnished the desired silyl enol ether, which after treatment with 1-benzyloxy-3-methyl-but-3-en-2-one and $SnCl_4$, gave the Mukaiyama-Michael adduct **30** in 61%. The α,β-enone **31** was obtained as a mixture of diastereoisomeres after cyclization of the 1,5-diketone in dilute solution (0.01 *M*) of potassium hydroxide in ethanol (83%) and elimination of the tertiary hydroxyl group in presence of thionyl chloride in pyridine with 83% yield. The oxime pivalate **32** was provided in 96% yield after the oxime formation using hydroxylamine followed by acylation with pivaloyl chloride. The aromatization and *N*-acylation of the intermediate was carried out using an acetyl chloride at 80$^{o}$C to furnish **33** in 64% yield[21]. The hydrogenolysis of the benzyl ether (99% yield), cyclization of the phenol with carbonyldiimidazole, and mildly basic aqueous workup ($NaHCO_3$) to remove the *N*-acyl group afforded the cyclic carbamate **34** in 94% yield. The deprotection in presence of HF-pyridine (95% yield), followed by mild oxidation to the aldehyde with tetrapropylammonium perruthenate and 4-methylmorpholine-*N*-oxide (TPAP-NMO, 82% yield) and *E*–selective olefination, gave the diene **35** in 81% yield. The key intermediate **36** was obtained in 80% yield by cationic cyclization in the presence of three equivalents of methanesulfonic acid and acetic acid as solvent at 19$^{o}$C. After *N*-protection using di-tert-butyl dicarbonate ($BOC_2O$) (96% yield) and excess of the methylmagnesium bromide ($CH_3MgBr$) to cleave the cyclic carbamate, followed by addition of the resulting dianion to a mixture of trifluoroacetic acid and triethylorthoformate, the natural product pseudopteroxazole **21** was achieved in 90% yield (Scheme 1)[21].

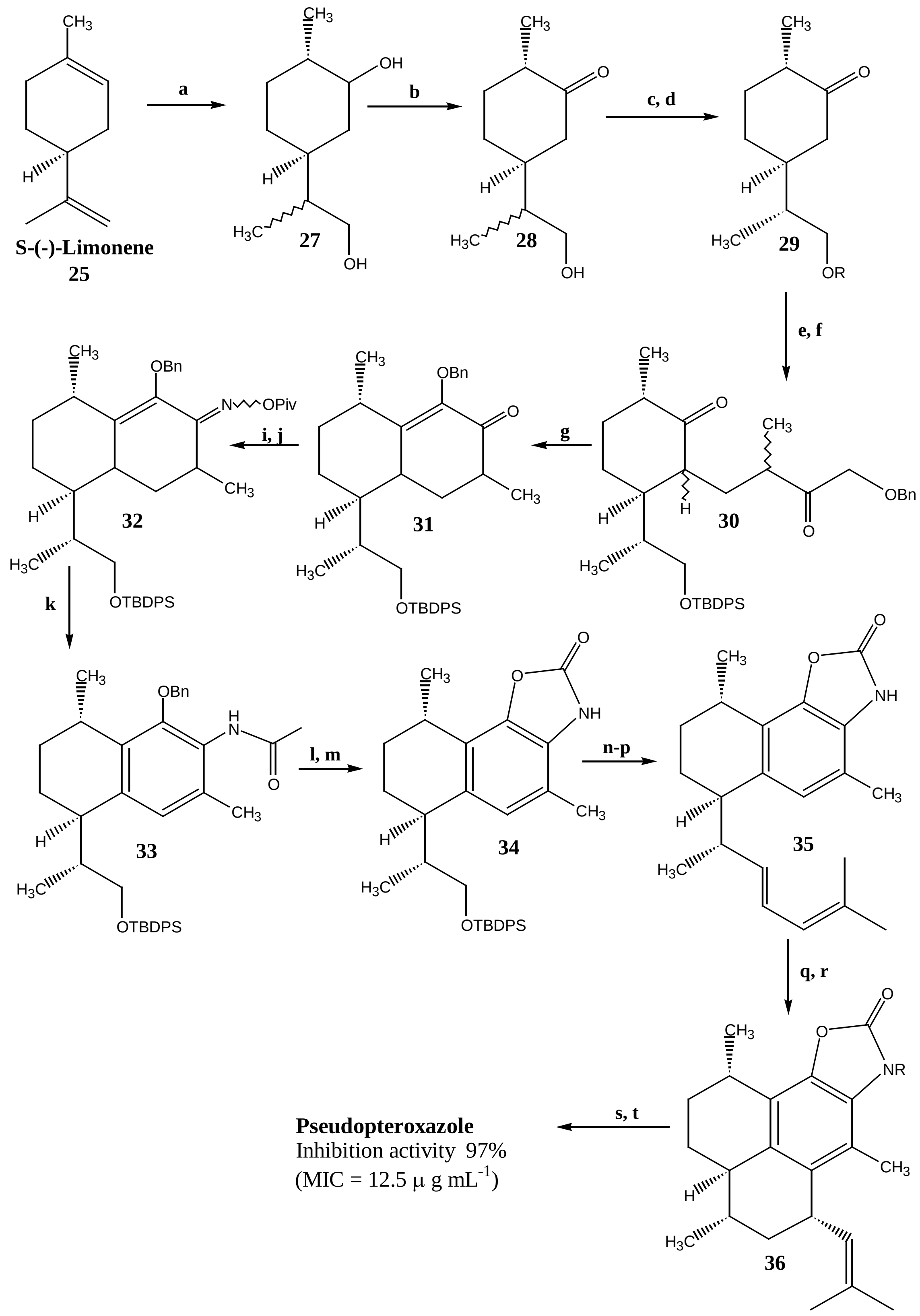

S-(-)-Limonene
25
a
27
b
28
c, d
29
e, f
30
g
31
i, j
32
k
33
l, m
34
n-p
35
q, r
36
s, t
Pseudopteroxazole
Inhibition activity 97%
(MIC = 12.5 μ g mL-1)

SCHEME 1

(a) Thexylboranes, alkaline peroxide oxidation, 92%; (b) NaClO, 86%; (c) AcOC($CH_3$)$CH_2$, lipase; (d) TBDPSCl, imidazole, DMF, 96%; (e) LDA, TMSCl, –78°C, 100%; (f) 1-benzyloxy-3-methyl-but-3-en-2-one, $SnCl_4$, $CH_2Cl_2$, –78°C, 61%; (g) KOH, EtOH, –10°C, 83%; (h) $SOCl_2$, py, 83%; (i) $NH_2OH$-HCl, py, 69%; (j) pivaloyl chloride, py, 96%; (k) acetylchloride, toluene, 80°C, 64%; (l) $H_2$, Pd$(OH)_2$/C, EtOH, 99%; (m) carbonyldiimidazole, $Et_3N$; then, aqueous $NaHCO_3$, 94%; (n) HF-py, 95%; (o) TPAP, NMO, 82%; (p) Wittig olefination, 81%; (q) $MeSO_3H$, AcOH, 18°C, 80%; (r) $BOC_2O$, DMAP, 96%; (s) MeMgBr, –78 to 23°C, then (t) TFA, HC(OEt)$_3$, 90%.

The phytosterol saringosterol **37** (Fig. 10), isolated by Timmermann and coworkers from the Chilean brown algae *Lessonia nigrescens Bory* (*Phaeophyta*, *Laminariales*)[22], showed potent anti-TB activity against the $H_{37}$Rv strain of *M. tuberculosis*. The 24*S* epimer of saringosterol presented MIC values of 0.125 μg/ml, eight times more active than their epimer 24*R* (1.0 μg/ml), indicating the importance of the hydroxyl position for biological activity. Due to its specifity, activity, and low toxicity, this class of compounds could be a good target for the development of new TB drugs[22].

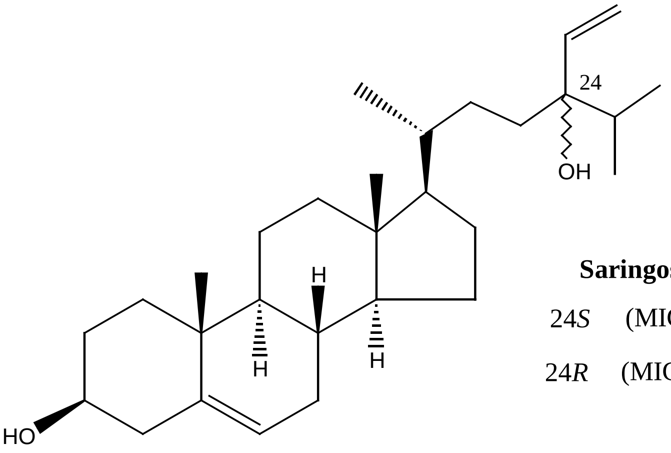

**Saringosterol (37)**

24*S* (MIC = 0.125 μ g mL$^{-1}$)

24*R* (MIC = 1.0 μ g mL$^{-1}$)

**FIGURE 10**

Berlinck and coworkers have isolated different compounds and the ingenamine G **38** from a cytotoxic and anti-TB, active methanol, crude extract of the marine sponge *Pachychalina* sp.[23]. The latter showed biological activity against *M. tuberculosis* (H37Rv) at 8 μg/ml (Fig. 11).

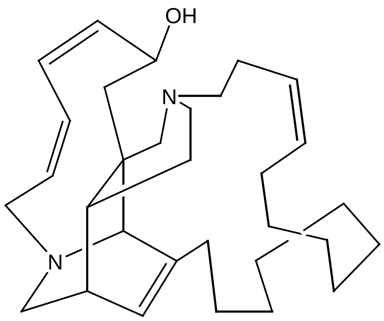

**Ingenamine G (38)**
(MIC = 8.0 μ g mL$^{-1}$)

**FIGURE 11**

Orabi and coworkers[24] identified potent anti-TB active in (+)-araguspongine C **39** (MIC = 3.94 μg/ml) (Fig. 12)[24], a natural product isolated from red sea specimens of *Xestospongia exigua*[25]. This class of compound possesses diverse important biological activities such as vasodilatation[26], cytotoxicity[27], antifungal[28], and vasoactive intestinal peptide inhibition activity[29].

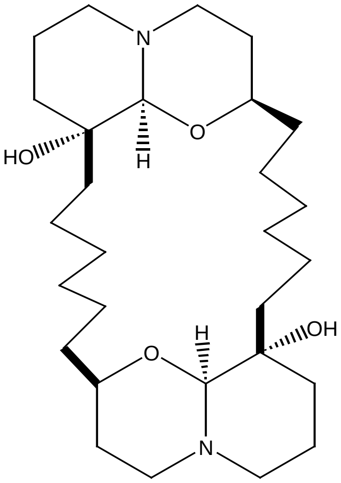

**(+)-Araguspongines C (39)**
(MIC = 3.94 μ g mL$^{-1}$)

**FIGURE 12**

Ecteinascidins 770 **40** and 786 **41** (Fig. 13) were isolate by Suwanborirux and coworkers from Thai tunicate *Ecteinascidia thurstoni*[30]. This class of compounds showed potent cytotoxic activity with the ecteinascidin 743 **42** under phase II clinical trials in the treatment of cancer[31]. Suwanborirux and his group found potent anti-TB activity against *M. tuberculosis* (H37Rv) in ecteinascidins 770 **40** and 786 **41** with MIC values of 0.13 and 2.0 μg/ml, respectively (Fig. 13)[30].

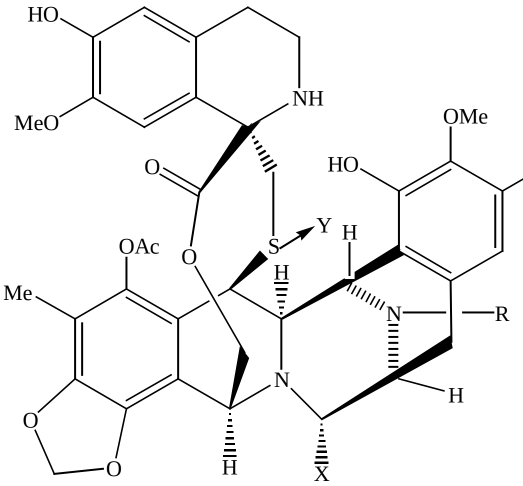

**Ecteinascidin**

**770 (40)** R = $CH_3$, X = CN, Y = none
(MIC = 0.13 μ g mL$^{-1}$)

**786 (41)** R = $CH_3$, X = CN, Y = O
(MIC = 2.0 μ g mL$^{-1}$)

**743 (42)** R = $CH_3$, X = OH, Y = none

**FIGURE 13**

Plubrukarn and coworkers isolated and tested different sesterpenes from Thai sponge B *Brachiaster* sp. against *M. tuberculosis* (H37Rv). As examples, the 12-deacetoxyscalarin 19-acetate **43** and the manoalide 25-acetate **44** (Fig. 14), with MIC values of 4 and 7 μg/ml, respectively, can be mentioned[32].

Bewley and coworkers isolated and identified new alkaloids **45**–**49** from Australian nonverongid sponge (Fig. 15)[33]. These compounds represent the first examples of inhibitors of a new mycobacterial enzyme S-conjugate amidase (MCA) that has been characterized from *M. tuberculosis* and *M. semegmatis*.

Due to the significant inhibitory activity of the alkaloid **47** against the MCA, Kende and coworkers were the first to synthesize the dibromotyrosine alkaloid **47** (Scheme 2)[34]. This synthesis used 4-hydroxyphenylpyruvic acid **50** as starting material, which was converted to the THP-oxime acid **51** in 90% yield, followed by the coupling with the protected 4-aminobutylguanidine **52** to afford the intermediate **53** in 87%. This intermediate was dibrominated to the dibromophenol amide **54** with *N*-bromosuccinimide (NBS) in 89% yield followed by the Mitsunobu coupling of the phenolic hydroxyl with 3-(*t*-butoxycarbonylamino)propanol, which furnished the compound **55** in 82% yield. The final step was carried out by removing all the protecting groups in the presence of 30% trifluoroacetic acid in dichlorometane to produce the target compound **47** as its bistrifluoroacetate salt in 82% yield[34].

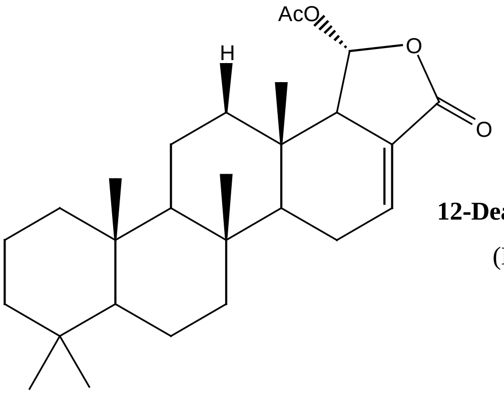

**12-Deacetoxyscalarin 19-acetate (43)**
(MIC =4.0 μ g mL$^{-1}$)

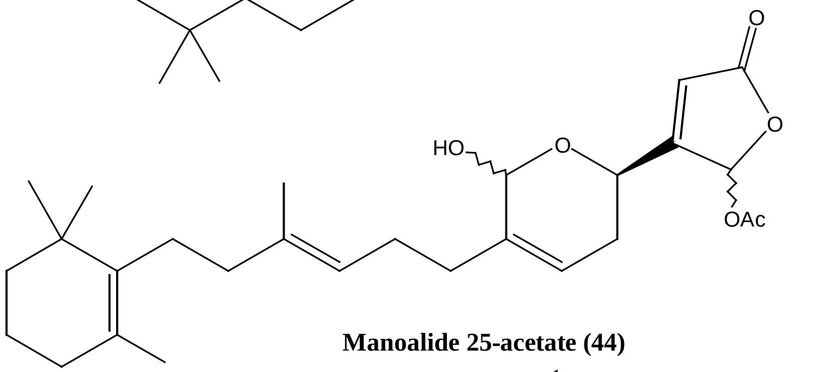

**Manoalide 25-acetate (44)**
(MIC =7.0 μ g mL$^{-1}$)

**FIGURE 14**

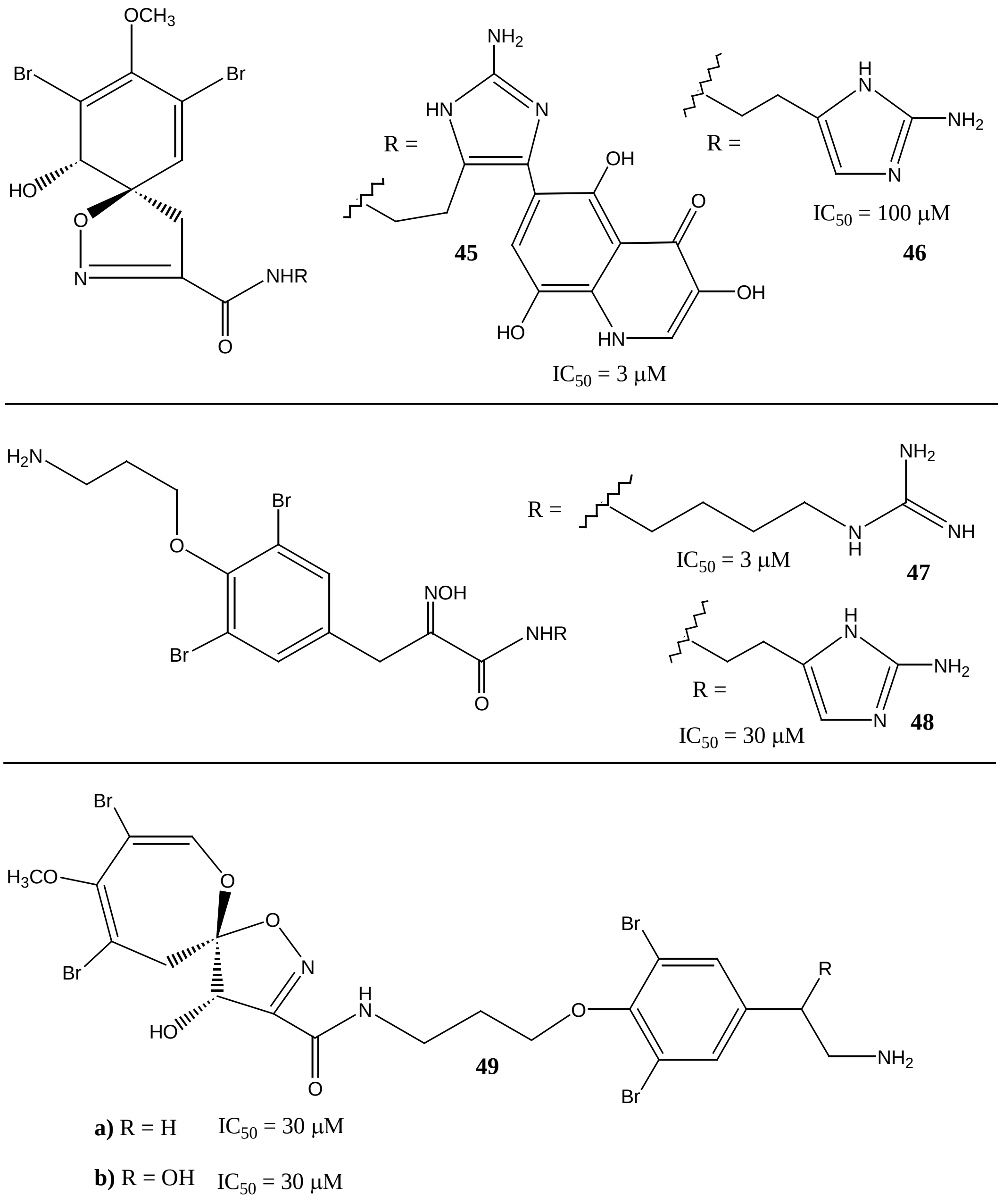

OCH3
Br
Br
HO
O
N
NHR
O
R =
NH2
HN
N
OH
O
OH
HO
HN
45
IC50 = 3 μM
R =
H
N
NH2
N
IC50 = 100 μM
46
H2N
Br
O
Br
NOH
NHR
O
R =
NH2
N
H
NH
IC50 = 3 μM
47
R =
H
N
NH2
N
IC50 = 30 μM
48
Br
H3CO
O
O
N
Br
HO
H
N
O
49
Br
R
O
Br
NH2
a) R = H IC50 = 30 μM
b) R = OH IC50 = 30 μM

**FIGURE 15**

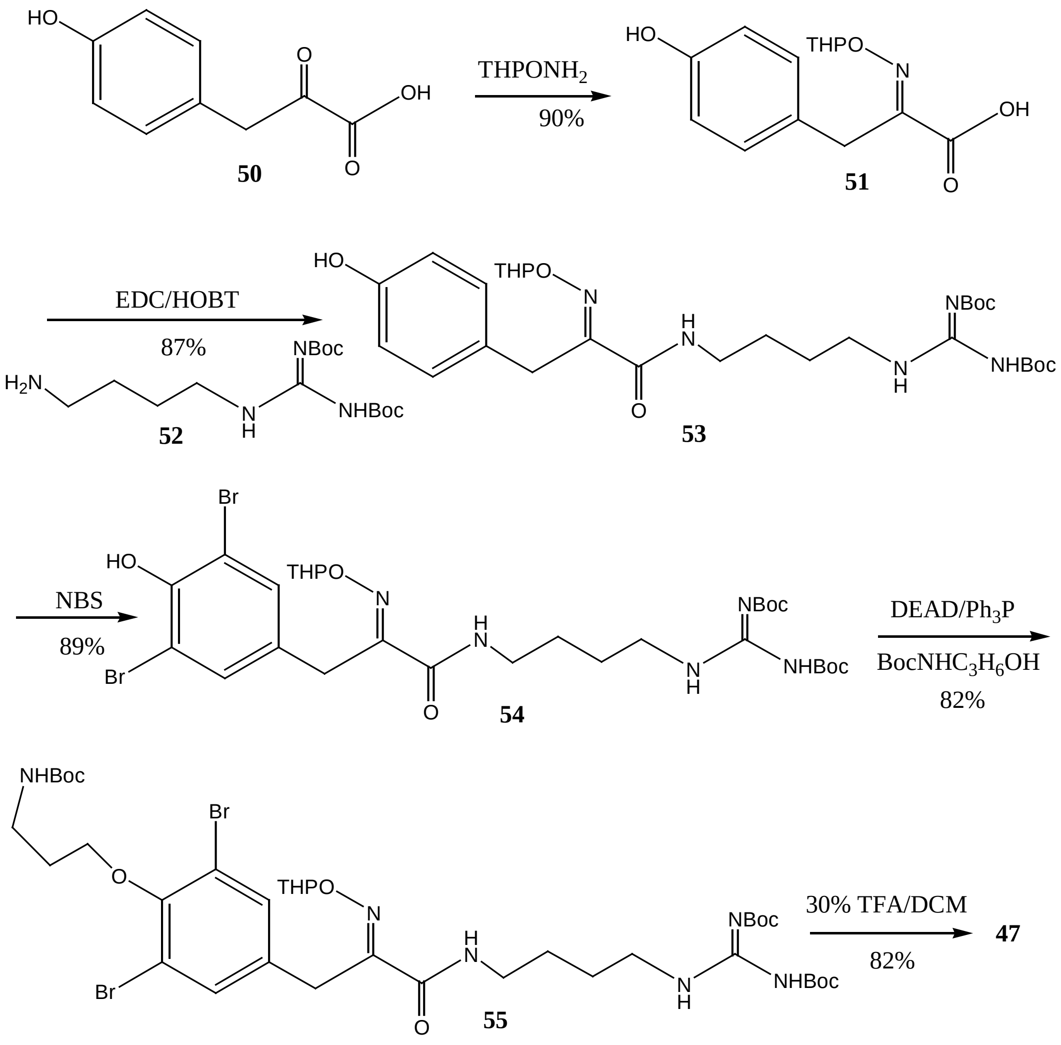

**SCHEME 2**

## CONCLUSION

To fight efficiently against the rapid spread of MDR TB strain against all major anti-TB drugs in the market, it is imperative have new drugs with fewer toxic side effects, improved pharmacokinetics properties, extensive and potent activity against Gram-positive and Gram-negative bacteria, including resistant strains, and drugs able to reduce the total duration of treatment. In this context, natural products, particularly from the ocean, offer unique chemical diversity with the potential to open a new era in TB treatment.

## ACKNOWLEDGMENTS

The author thanks Solange M., S.V. Wardell, and James L. Wardell for reading this manuscript.
